# Anti-melanoma activity of Forsythiae Fructus aqueous extract in mice involves regulation of glycerophospholipid metabolisms by UPLC/Q-TOF MS-based metabolomics study

**DOI:** 10.1038/srep39415

**Published:** 2016-12-19

**Authors:** Jiaolin Bao, Fang Liu, Chao Zhang, Kai Wang, Xuejing Jia, Xiaotong Wang, Meiwan Chen, Peng Li, Huanxing Su, Yitao Wang, Jian-Bo Wan, Chengwei He

**Affiliations:** 1 State Key Laboratory of Quality Research in Chinese Medicine, Institute of Chinese Medical Sciences, University of Macau, Macao 999078, China

## Abstract

Metabolomics is a comprehensive assessment of endogenous metabolites of a biological system in a holistic context. In this study, we evaluated the *in vivo* anti-melanoma activity of aqueous extract of Forsythiae Fructus (FAE) and globally explored the serum metabolome characteristics of B16-F10 melanoma-bearing mice. UPLC/Q-TOF MS combined with pattern recognition approaches were employed to examine the comprehensive metabolic signatures and differentiating metabolites. The results demonstrated that FAE exhibited remarkable antitumor activity against B16-F10 melanoma in C57BL/6 mice and restored the disturbed metabolic profile by tumor insult. We identified 17 metabolites which were correlated with the antitumor effect of FAE. Most of these metabolites are involved in glycerophospholipid metabolisms. Notably, several lysophosphatidylcholines (LysoPCs) significantly decreased in tumor model group, while FAE treatment restored the changes of these phospholipids to about normal condition. Moreover, we found that lysophosphatidylcholine acyltransferase 1 (LPCAT1) and autotaxin (ATX) were highly expressed in melanoma, and FAE markedly down-regulated their expression. These findings indicated that modulation of glycerophospholipid metabolisms may play a pivotal role in the growth of melanoma and the antitumor activity of FAE. Besides, our results suggested that serum LysoPCs could be potential biomarkers for the diagnosis and prognosis of melanoma and other malignant tumors.

Cancer has been a leading cause of death worldwide, both in developed and developing countries^1^. Melanoma is one of the most aggressive malignancies in all the cancer types, which has a rapidly increasing incidence for decades^2^. Conventional chemotherapeutic agents often show limited therapeutic efficiency on melanoma in clinical practice^3^ because of its resistance to chemo- and radiotherapy^4^. Therefore, it is imperative to develop new therapeutic approaches to treat this malignancy. In recent years, the antitumor potential of natural products, especially herbs from formulas of traditional Chinese medicine (TCM), which has been widely prescribed by experienced TCM practitioners for the prevention and treatment of diseases in Oriental countries for thousands of years, attracted increasing attention due to their multiple targets and less side effects. TCM herbs are being considered to be a valuable source of potential novel anticancer agents^5^,^6^.

TCM herbs or formulas contain hundreds even thousands of compounds, however, most of their chemical properties and biological activities have not been identified yet. Although the preventive and therapeutic efficacy of TCM herbs or formulas have been extensively verified in clinical practice, the molecular mechanisms of their actions are still largely unknown due to the complex chemical components, multiple molecular targets, and the lack of effective scientific research approaches. Since the most prominent characteristic of TCM is to use holistic integrated measures to examine and treat a symptom or disease^7^, it requires holistic systematic approaches, such as genomic, proteomic or metabolomics tools, to comprehensively investigate the complex efficacies and molecular mechanisms of TCM^8^.

Metabolomics is a newly arisen branch of systems biology, which aims to systematically analyze all metabolites in a biological sample. Its significant potential has been recently demonstrated in many fields for disease diagnosis, drug development, drug safety and toxicology evaluation, therapeutic effect evaluation and biomarker identification^9^,^10^,^11^. Compared with conventional methods for assessing therapeutic effects and mechanisms of TCM, the obvious advantages of metabolomics is that it could provide a global unbiased analysis of metabolites in an organism which coincides with the holism concept of TCM^12^. In fact, metabolomics has been playing important roles in evaluating the effect of TCM on many diseases and revealing its molecular basis through a global view of an organism^13^.

Forsythiae Fructus, Lianqiao in Chinese, the dry fruit of *Forsythia suspensa* (Thunb.) Vahl. of Oleaceae family, has been widely used in TCM for over thousands of years. Forsythiae Fructus has the bitter, cool, and slightly acrid characteristics and is classified as a typical heat-clearing herb according to TCM theory^14^. In traditional clinical applications, Forsythiae Fructus is commonly used to treat boils, carbuncles, mumps, tonsillitis, urinary tract infections, allergic rashes, colds or flu-like symptoms (e.g. fever, chills, and headache), etc.^15^,^16^. We previously reported that Forsythiae Fructus aqueous extract (FAE) exhibited potent antitumor activity *in vitro* and *in vivo*^17^. However, the precise molecular mechanisms have not been well elucidated. Here, we examined the antitumor effects of FAE on B16-F10 melanoma and its possible molecular mechanisms using metabolomics approaches, in which ultra-performance liquid-chromatography/quadrupole time of flight mass spectrometry (UPLC/Q-TOF MS) method was employed to obtain the comprehensive serum metabolic signatures of experimental mice, and pattern recognition approaches, including principal component analysis (PCA), partial least squares-discriminant analysis (PLS-DA) and orthogonal PLS-DA (OPLS-DA), were used to identify the differentiating metabolites. We found that the regulation of glycerophospholipid metabolism may perform an important role in the tumor growth of melanoma and antitumor effects of FAE, and the serum LysoPCs could be potential biomarkers for the diagnosis and prognosis of melanoma and efficacy evaluation of antitumor treatment.

## Results

### Antitumor effect of FAE on B16-F10 melanoma in C57BL/6 mice

**Figure 1:**
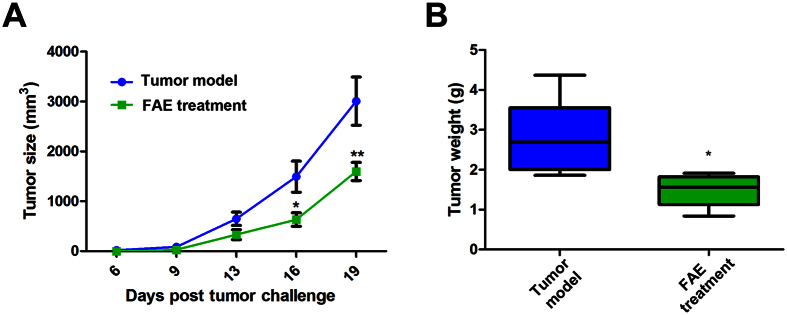
Antitumor effect of FAE (10 g/kg) on B16-F10 melanoma *in vivo*. (**A**) Tumor size was measured twice a week from day 6. When the tumor size is greater than 2500 mm^3^, the mice were sacrificed. (**B**) Tumor weight was measured on the final day (day 21) of the experiment. Data are means ± SEM, *n* = 6. The *in vivo* antitumor effects of FAE were analyzed by two-way ANOVA method. *p < 0.05, and **p < 0.01 compared to the tumor model group.

We used a transplantation tumor mouse model to determine the antitumor activity of FAE *in vivo*. Murine melanoma B16-F10 cells were transplanted s.c. into mice on day 0 and FAE was given at concentrations of 10 g/kg by oral gavage on the same day and every two days after that. As shown in Fig. 1A, the effect of FAE on tumor growth was monitored. Significant inhibition of tumor growth was recorded after day 16 and day 19 in the FAE treatment group, respectively. FAE (10 g/kg) treatment group had a 47.2 ± 14.4% inhibitory rate on tumor growth in mice comparing tumor weight with the control group (Fig. 1B). These results demonstrated that FAE exhibited a significant antitumor activity against B16-F10 melanoma *in vivo*.

### Validation of UPLC/Q-TOF MS method

QC sample was prepared by mixing the aliquots of each tested serum sample, which provided a representative sample of all components in a relatively average concentration. QC sample served as technical replicates throughout the data set to validate system stability and reproducibility of UPLC/Q-TOF MS method. QC sample was analyzed 3 times at the beginning of the analysis and once every 5 tested samples to further assess the stability of the method. Three typical ion pairs (1.9414 min_m/z 307.0562, 7.3447 min_m/z 494.3182, 7.59 min_m/z 1039.7468) in positive ion mode and three ion pairs (9.1131 min_m/z 480.3, 3.5077 min_m/z 172.9556, 7.592 min_m/z 524.2744) in negative ion mode were selected for method validation. The relative standard deviation (RSD) of ion intensity, retention time and m/z were 2.69–8.49%, 0–0.17%, 0.00006–0.00027%, respectively. These results demonstrated that the UPLC/Q-TOF MS method had excellent stability and reproducibility.

### Analysis of metabolic profiles in serum by UPLC/Q-TOF MS

**Figure 2:**
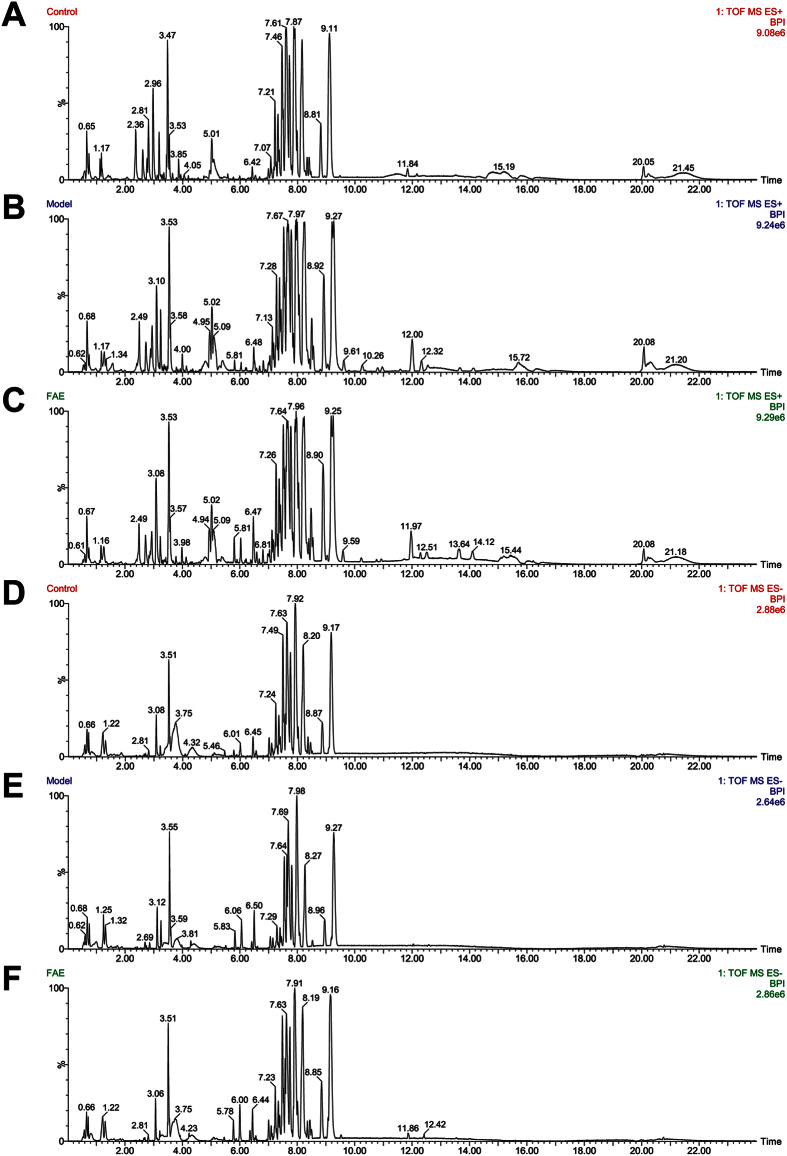
Typical UPLC-Q/TOF MS BPI chromatograms of serum samples. (**A–C**) Positive ESI mode, (**D–F**) Negative ESI mode, (**A,D**) Control group, (**B,E**) Tumor model group, (**C,F**) FAE treatment group.

In order to investigate the endogenous metabolites changes in serum of melanoma-bearing mice with and without FAE treatment, the metabolic profiles of serum samples from control, tumor model and FAE treatment groups were characterized by UPLC/Q-TOF MS in both positive and negative ion modes. The typical UPLC-Q/TOF MS base peak intensity (BPI) chromatograms of representative sample collected from each group were shown in Fig. 2. A data matrix was generated after pre-processing consisting of the retention, m/z value and the normalized peak area. 2515 pairs of RT-m/z in positive ion mode and 564 pairs of RT-m/z in negative ion mode in peak list were observed.

### Multivariate statistical analysis

**Figure 3:**
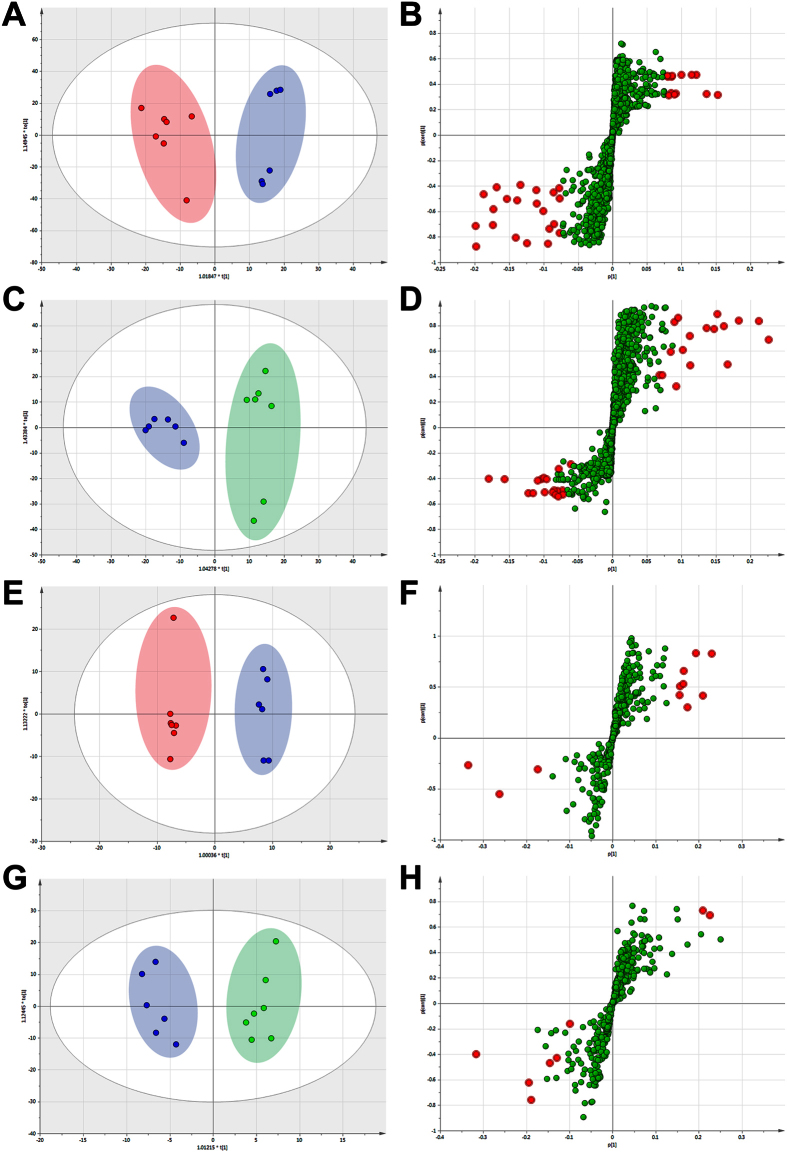
OPLS-DA score plot and its corresponding S-plot based on UPLC-MS profiling data of serum samples. (**A**) OPLS-DA score plot in control group (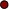) and tumor model group (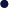) (**B**) S-plot in control group and tumor model group detected in positive ion mode. (**C**) OPLS-DA score plot in tumor model group and FAE treatment group (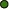). (**D**) S-plot in tumor model group and FAE treatment group detected in positive ion mode. (**E**) OPLS-DA score plot in control group and tumor model group. (**F**) S-plot in control group and tumor model group detected in negative ion mode. (**G**) OPLS-DA score plot in tumor model group and FAE treatment group. (**H**) S-plot in tumor model group and FAE treatment group detected in negative ion mode. The variables with VIP >3.0 were highlighted with red filled circle (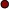).

The peak list containing the retention time, m/z, and peak area of each sample were imported into the SIMCA-P software for multivariate pattern recognition analysis. Principal component analysis (PCA) approach provided unsatisfactory separation of data between the control and melanoma model groups (data not shown). In order to obtain better discrimination between the control and model group, OPLS-DA approach was applied. As shown in Fig. 3A,E, each point represented a tested serum sample. The loading plot of OPLS-DA clearly separated tested samples into two blocks according to their metabolic profiles of different groups in both positive and negative ion modes. These results indicated that the biochemical metabolites in serum were perturbed significantly in melanoma model group comparing with control group.

In the corresponding S-plot, each point represented an ion RT-m/z pair. The variables with higher *p* and *p* (corr) values were more important to discriminate two groups which always shown in upper-right and lower-left quadrants of the S-plot. As shown in Fig. 3B,F, the variables with variable importance in projection (VIP) values exceed 3.0 were highlighted with red filled circle. These red points were relevant variables to separate the control group and melanoma model group.

The ion RT-m/z pairs also showed significant difference in abundance between the model group and FAE treatment group through OPLS-DA analysis (Fig. 3C,G). In the corresponding S-plot, the variables with VIP value exceed 3.0 were also marked with red filled circle (Fig. 3D,H). The variable points in upper-right represented the relative intensity of ion RT-m/z pairs in FAE group which were increased comparing with model group. Combining the results of the OPLS-DA analysis of control, model groups and FAE treatment groups, 25 ion RT-m/z pairs were selected as potential biomarkers from the list of red filled circle in positive and negative mode.

### Metabolomics analysis of intervention effect of FAE

**Figure 4:**
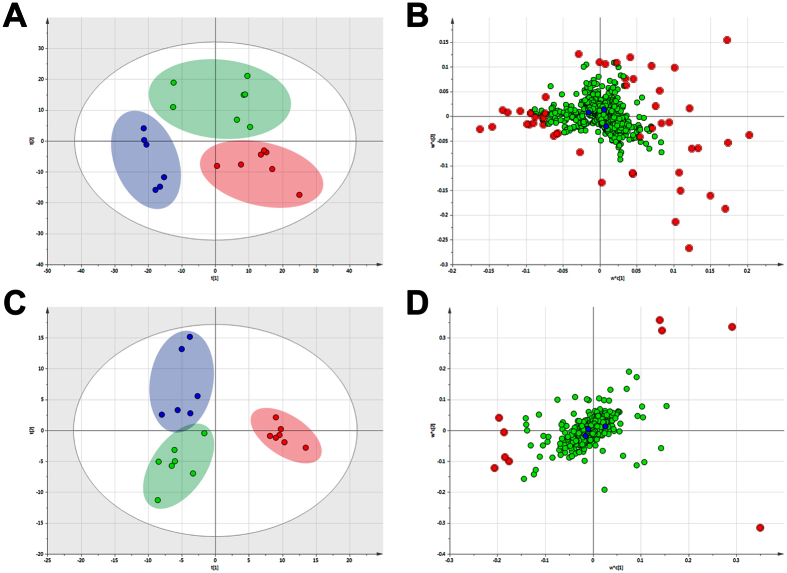
PLS-DA score plot and its corresponding loading plot based on UPLC-MS profiling data of serum samples. (**A**) PLS-DA score plot in control group (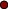), tumor model group (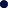) and FAE treatment group (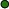) detected in positive ion mode, with fitting and predictive performance (3 latent variables, *R*^*2*^*X* = 0.478, *R*^*2*^*Y* = 0.825, *Q*^*2*^ = 0.36). (**B**) Loading plot in three groups detected in positive ion mode. (**C**) PLS-DA score plot in control, tumor model and FAE treatment groups detected in negative ion mode, with fitting and predictive performance (2 latent variables, *R*^*2*^*X* = 0.248, *R*^*2*^*Y* = 0.808, *Q*^*2*^ = 0.313). (**D**) Loading plot in three groups detected in negative ion mode. The variables with VIP >3.0 were highlighted with red filled circle (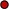).

We evaluated the intervention effect of FAE on the metabolic profile through PLS-DA analysis which could discriminate groups according to their relative concentrations of metabolites. As shown in Fig. 4, the obvious separation among the control, tumor model and FAE treatment groups was observed in the PLS-DA score plots in both positive and negative ion modes according to the differential metabolites in each group. The cluster of model group was the furthest from that of control group which suggested that the metabolic profile was intensely perturbed by the influence of melanoma growth. Similarly, a clear separation between model and FAE treatment groups was obtained, indicating that the serum metabolic profile was significantly changed by FAE treatment. Interestingly, it is easy to see that, in the direction of the first principal component, the cluster of FAE treatment group was much closer to control group than that of model group in the positive ion mode. These results revealed that FAE treatment at the dose of 10 g/kg significantly restored the disturbed serum metabolic profile in melanoma-bearing mice to a relatively normal condition.

### Chemical identification and metabolic pathway analysis

The retention time, precise molecular mass and MS/MS data of the 25 variables provided by UPLC/Q-TOF MS analysis were used to identify the chemical structures of metabolites by the procedures as described in the method part. Seventeen variables, 16 in positive mode and one in negative mode, were found to be distinct compounds, and 13 of them were chemically identified. Table 1 shows the identification results and content changes of the 17 metabolites in mice serum.

**Figure 5:**
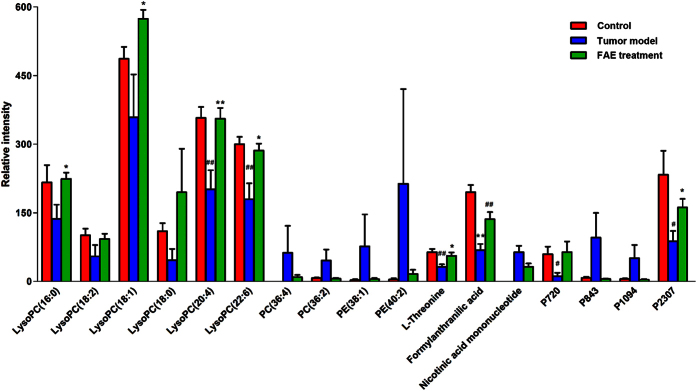
Changes in the relative intensity of target metabolites. The target metabolites identified by S-plot in control, tumor model and FAE treatment groups. ^#^*p* < 0.05, ^##^*p* < 0.01 compared to the control group; *p < 0.05, **p < 0.01 compared to the tumor model group, by One-way ANOVA with Tukey post hoc analysis.

The relative intensity changes of 17 metabolites in control, model and FAE treatment groups were statistically analyzed by one-way ANOVA and shown in Fig. 5. In tumor model group, LysoPCs were down-regulated significantly, while they were restored to about normal levels by FAE treatment, indicating that FAE could significantly reverse the decreased LysoPC levels induced by melanoma growth. Similar results were observed in the intensity changes of L-threonine, formylanthranilic acid, and two unknown metabolites P720 and P2370. In addition, four other phospholipids, including two phosphatidylcholines (PCs) and two phosphatidylethanolamines (PEs), and two unknown metabolites P843 and P1094 showed a strong but opposite trend, though there were no significant differences.

**Figure 6:**
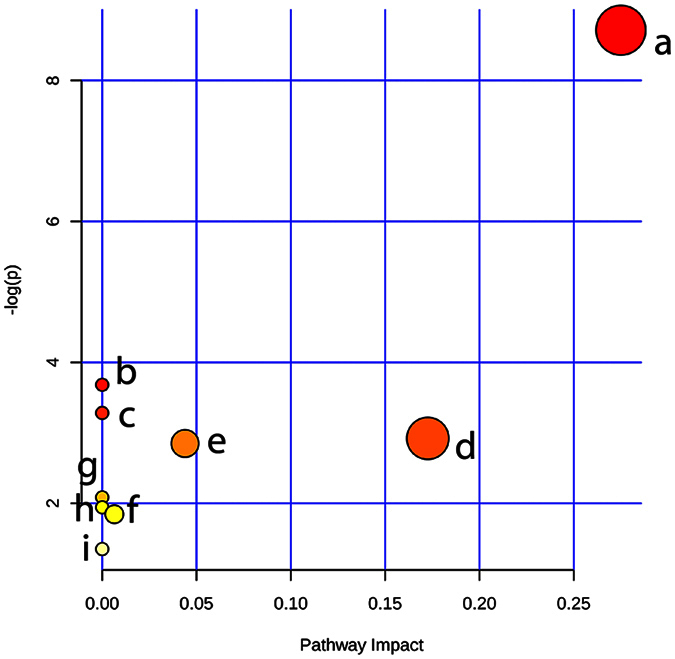
Summary of pathway analysis with MetaboAnalyst 3.0. Each point represents one metabolic pathway; the size of dot is in positive correlation with the impaction of the metabolic pathway.

In order to explore the most relevant metabolic pathways influenced by melanoma growth and FAE treatment, MetaboAnalysis 3.0 was used based on the significantly different metabolites. The results showed that nine metabolic pathways were affected by tumor insult and FAE treatment (Fig. 6). The involved pathways with higher impact value are more significantly influenced. Those pathways with impact value more than 0.1 were considered as the significantly relevant pathways. Among the nine pathways, glycerophospholipid metabolism got the highest impact value of 0.275 in this study (Table 2), and was suggested to be most responsible for the melanoma growth and antitumor effect of FAE. In addition, there were three hits (KEGG ID C04230, C00157, and C00350) for glycerophospholipid metabolism and only one hit for nicotinate and nicotinamide metabolisms. Moreover, 10 out of 13 identified metabolites were involved in glycerophospholipid metabolism (Table 3), which further confirmed glycerophospholipid metabolism was the prominently affected pathway by melanoma insult and FAE treatment.

### Effect of FAE on the expression of LPCAT1 and ATX

**Figure 7:**
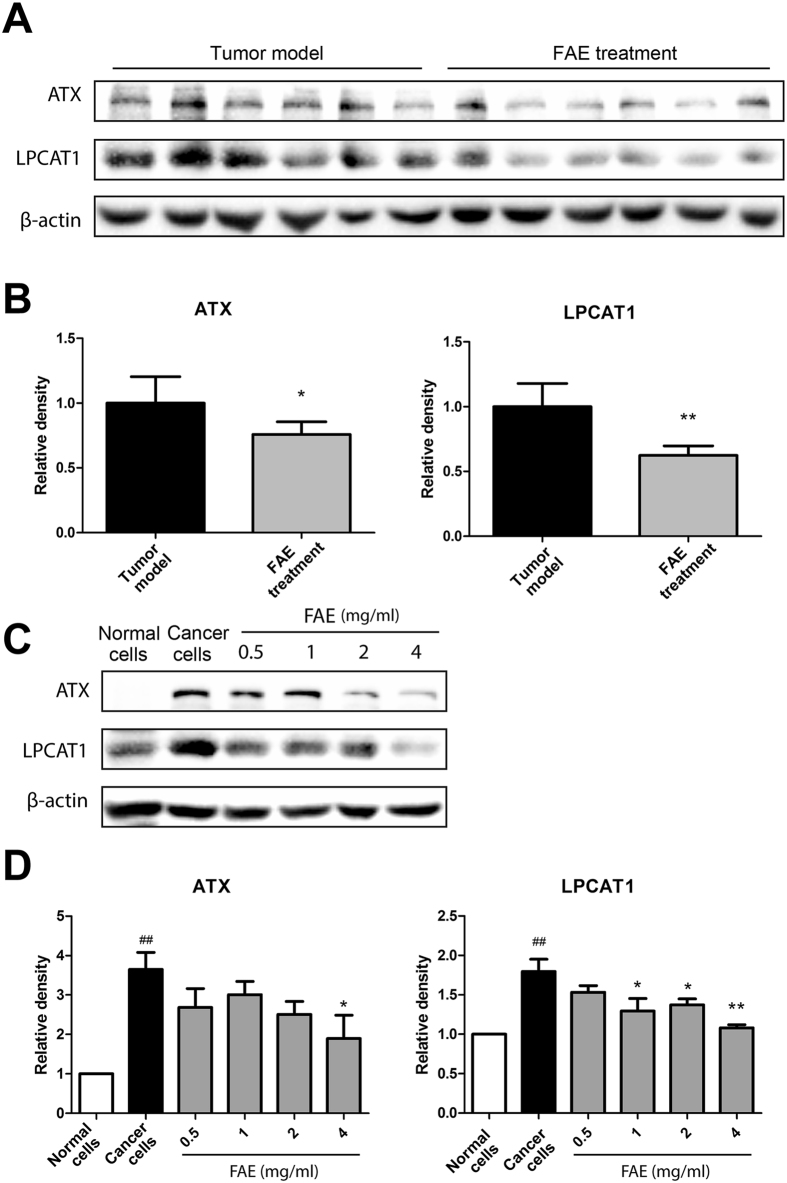
Effects of FAE on expression of LPCAT1 and ATX in tumor tissues and cancer cells. Western blotting was used to determine the protein expression of LPCAT1 and ATX in tumor tissues with or without FAE treatment (**A**), NIH 3T3 normal cells and B16-F10 cancer cells with or without FAE treatment (**C**). (**B,D**) The density of each band was quantified by Quantity One Software, and the relative density ratio of each protein was calculated accordingly. β-actin was used as the internal reference. Data are expressed as means ± SD. *p < 0.05, **p < 0.01, compared to tumor model group or B16-F10 cancer cell group. ^##^*p* < 0.01, compared to NIH 3T3 normal cell group.

In order to verify the results obtained from metabolomics analysis, we examined the protein expression levels of lysophosphatidylcholine acyltransferase 1 (LPCAT1) and autotaxin (ATX), which are key enzymes participating in glycerophospholipid metabolisms. As shown in Fig. 7A,B, LPCAT1 and ATX were highly expressed in tumor tissues and FAE significantly decreased the expression of these two enzymes. In addition, FAE treatment markedly inhibited the expression of LPCAT1 and ATX in B16-F10 cells (Fig. 7C,D). These results suggested that the increased LysoPCs and decreased PCs in FAE-treated groups were owing to, at least partially, down-regulated LPCAT1 and ATX expression in cancer cells by FAE.

## Discussion

Melanoma is one of the most aggressive malignancies worldwide and its incidence and mortality are still increasing in recent years^18^. There is no effective therapeutic approach for the treatment of melanoma so far due to the frequent occurrence of drug resistance and metastasis in this malignancy. In current years, increasing attention has been paid to natural products, especially TCM herbs, because of their multi-target and low toxicity^5^. However, the complex activities and mechanisms of TCM herbs are still largely unknown due to the lack of efficient research approaches. Metabolomics provides a global unbiased analysis of metabolites in an organism which coincides with the holism concept of TCM. In the present study, we used metabolomics approaches to explore the antitumor effect and molecular mechanisms of TCM herbs. Our results demonstrated that the Forsythiae Fructus water extract FFA could markedly inhibit the growth of B16-F10 melanoma in C57BL/6 mice. Importantly, we revealed that the significant changes in serum metabolic profile of tumor-bearing mice were greatly restored after FAE treatment. In particular, the antitumor activity of FAE is closely associated with the regulation of glycerophospholipid metabolism.

Metabolomics provides a robust tool for assessing the effect of TCM herbs or formulas, as well as understanding the mechanisms. The integration strategy and approaches of metabolomics coincide with the holistic concept and practices of TCM, suggesting that it could have the potential and advance for TCM research. Recently, there have been increasing studies applying metabolomics tools in the investigation of efficacy, molecular mechanisms and potential biomarkers in the area of TCM^12^. Liu *et al*. reported the protective effect of *panax notoginseng* saponins (PNS) on alcoholic liver injury, which was based on the metabolomics approach. In their study, a urinary metabolomics method based on LC-MS and pattern recognition analysis was used to evaluate the effect of PNS on chronic alcohol-induced liver injury in mice. The effect of PNS on the changes in metabolic profile induced by chronic alcohol exposure was examined by PCA and OPLS-DA pattern recognition analysis^19^. In our study, the similar approach was used to measure the metabolites in serum samples and assess the antitumor effect of herb extracts. As expected, we found that the FAE treated group was clearly separated from the tumor model group and much closer to the control group in the direction of the first principal component. In addition, the changes of these metabolites in model group could be significantly reversed by herb extract treatment. Metabolomics is also an effective tool for the investigation of the underlying molecular mechanisms of TCM. An *et al*.^20^ comprehensively explored the possible mechanisms of the hepatoprotective effects of Zhi-Zi-Da-Huang decoction, a TCM formula, through an NMR-based metabolomics study. The results showed that a series of differentially expressed metabolites in plasma and liver were identified and suggested that the protective effects of the decoction on liver injury may be exerted through mitigating the impairment of energy and materials metabolisms, lipid peroxidation, permeability change of membrane and oxidative stress induced by alcohol. In the current study, 2515 RT-m/z ion pairs in positive and 564 in negative ion mode were observed by UPLC/MS analysis. Then, 25 of them with VIP values greater than 3.0 were selected as potential biomarkers after multivariate statistical analysis. Finally, 13 distinct components (Tables 1 and 3) that were correlated to the antitumor effect of FAE were identified, including lipids, amino acid and co-enzyme. Notably, 10 of the identified metabolites were phospholipids, such as LysoPCs, PCs and PEs. We observed that levels of LysoPCs, including LysoPC (16:0), LysoPC (18:0), LysoPC (18:1), LysoPC (18:2), LysoPC (20:4) and LysoPC (22:6), were decreased significantly in melanoma model group comparing with those in control group, and restored significantly in the FAE treatment group (Fig. 6). Meanwhile, the levels of PC (36:4) and PC (36:2) were increased in the model group and recovered in FAE treatment group. These results suggest that the antitumor activity of FAE could be attributed to the regulation of phospholipid metabolisms in melanoma by FAE.

LysoPCs are major plasma lipids and has been recognized as an important cell signaling molecule converted from phosphatidylcholine under physiological conditions catalyzed by phospholipase A_2_. LysoPCs transport glycerophospholipid components such as fatty acids, phosphatidylglycerol and choline between tissues^21^. The lower levels of LysoPCs may reflect a higher metabolism rate in cancer patients. LysoPC is an important intermediate in the degradation and biosynthesis of phosphatidylcholine (PC) which has been an important biomarker in cancer diagnosis, such as breast cancer^22^, lung cancer^23^, ovarian cancer^24^,^25^, gastric cancer^26^, colorectal cancer^27^ and melanoma^28^. LysoPC levels were shown to be decreased in the serum or urine of these cancer patients. A prospective metabolomics study showed that higher levels of LysoPC 18:0 were related to a lower risk of common cancers. In contrast, higher levels of PC were associated with increased cancer risk^29^. Moreover, Jantscheff *et al*. found that LysoPC pretreatment reduced B16-F10 melanoma cell adhesion *in vitro* and inhibited metastasis-like lung invasion *in vivo*^28^. Therefore, low levels of LysoPCs and high levels of PCs could be considered as cancer risk factors.

**Figure 8:**
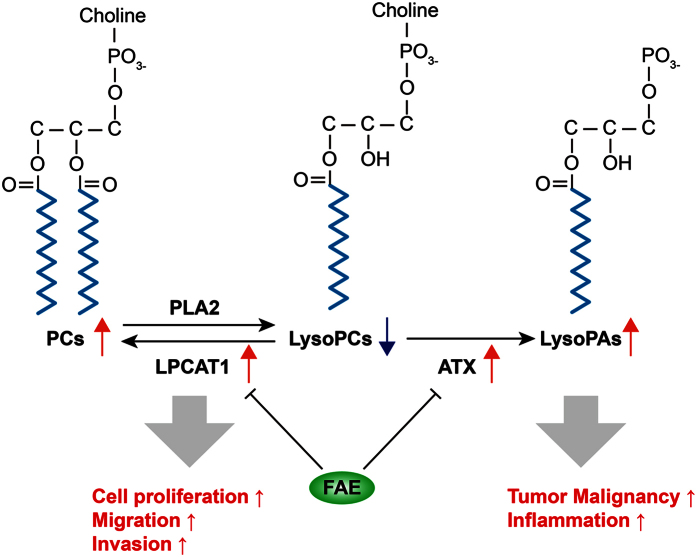
Schematic diagram showing the roles of LPCAT1 and ATX in LysoPC metabolism in cancer cells and the regulation by FAE. LPCAT1, Lysophosphatidylcholine acyltransferase 1; ATX, Autotaxin; PLA2, Phospholipase A2; LysoPAs, Lysophosphatidic acids.

LysoPCs can be an important precursor for the synthesis of PC by the action of LPCAT1 which is overexpressed in several cancers, and increased incorporation of PCs into cell membranes may facilitate the proliferation, adhesion, and motility of cancer cells^26^,^30^. LPCAT1 has been an independent predictor of early tumor recurrence and represents a novel prognostic biomarker for breast cancer, gastric cancer and prostate cancer^26^,^31^,^32^. LysoPCs are also a substrate of lysophospholipase D (lysoPLD)^33^,^34^. ATX, a secreted lysoPLD, converts LysoPCs to lysophosphatidic acid (LysoPA), which have been implicated in cancer development^35^. Both ATX and LysoPA have been potential biomarkers for cancer diagnosis. Overexpression of ATX and LysoPA has been observed in several cancers, including glioblastoma, thyroid carcinomas, and renal cell carcinoma^27^. Since both ATX and LysoPA receptor knockout mice show lower cancer risk, overexpression of ATX and LysoPA receptors has been proposed to be a common feature of several cancers^29^. In our results, we found that LPCAT1 and ATX were highly expressed in melanoma tissues and B16-F10 cancer cells (Fig. 7), which is consistent with previous reports. The up-regulation of LPCAT1 and ATX expression could be responsible for the decreased LysoPCs and increased PCs in the plasma of B16-F10 melanoma-bearing mice. Moreover, the expression of LPCAT1 and ATX in melanoma tissues and cancer cells were markedly inhibited by FAE treatment (Fig. 7), suggesting that the regulation of glycerophospholipid metabolisms by FAE was through, at least partially, down-regulating LPCAT1 and ATX expression in cancer cells (Fig. 8).

Previously, LysoPCs had been reported as a pro-inflammatory lipid. The pro-inflammatory action of LysoPCs may be largely due to the generation of reactive oxygen species or nitric oxide in various types of cells^36^,^37^,^38^,^39^. In contrast, studies reported that some LysoPCs could exhibit anti-inflammatory effect by inhibiting the formation of pro-inflammatory leukotrienes and cytokines *in vivo*^40^,^41^. It was found that the decreased LysoPC levels were related to chronic inflammation in obesity^42^. Meanwhile, the antiatherogenic effects of LysoPCs in acute and chronic inflammation have been observed^43^. However, it remains unclear whether LysoPCs exert pro-inflammatory or anti-inflammatory effects in the context of melanoma. In our previous study, we found that the antitumor effect of FAE was associated with the anti-inflammatory and antioxidant properties of FAE^44^. Together with the data of current study, we speculate that LysoPCs may function as an anti-inflammatory mediator. Further studies are required to clarify this conjecture. Our study and others also strongly suggest that dysregulation of LysoPC metabolism may play a crucial part in tumorigenesis and the LysoPC profiles in urine or blood could be potential biomarkers for diagnosis, prognosis, and treatment efficacy evaluation of tumor.

Taken together, the present study demonstrated that UPLC/Q-TOF MS-based metabolomics approaches could be useful tools for the investigation of the antitumor efficacy and its complex molecular mechanisms of TCM herbs. Using this technology, we effectively found that FAE exhibited a potent antitumor activity and rectified the disturbed metabolic profile by tumor insult. Notably, FAE restored the down-regulated LysoPCs and up-regulated PCs in B16-F10 melanoma-bearing mice, indicating that the anti-melanoma activity of FAE might be through, at least partially, the modulation of glycerophospholipid metabolisms. In addition, our results suggest that serum LysoPCs and PCs are potential biomarkers for the diagnosis and prognosis of melanoma and other malignant tumors, and efficacy evaluation of antitumor treatment.

## Materials and Methods

### Chemicals and reagents

HPLC-grade acetonitrile (CH3CN) and formic acid were obtained from Merck KGaA (Darmstadt, Germany). HPLC-grade methanol was purchased from Sigma-Aldrich (St. Louis, MO, USA). Water was purified by the Milli-Q water purification system (Millipore, Bedford, MA, USA). All other reagent were of analytical grade.

### Preparation of FAE aqueous extract

Dried Forsythiae Fructus was purchased from Sichuan Neautus Traditional Chinese Medicine Co., Ltd (Chengdu, Sichuan, China). Forsythiae Fructus aqueous extract (FAE) was prepared by adding 100 g of dried herbal powder to 1000 ml distilled water to form slurry, bringing the mixture to a boiler and simmering at 80 °C for 60 minutes. After cooling down, the aqueous layer was carefully decanted off of the residual solids. The nominal concentrations of Forsythiae Fructus aqueous extract were relative to the quantity of the dried herbal powder. The extract was concentrated to 1/20 of the original volume. The nominal concentration of the concentrated FAE was 2 g/ml. The extract was stored at −20 °C until use. Generally, the extracts were tested within a month after preparation.

### Animals and treatments

C57BL/6 mice were purchased from The Chinese University of Hong Kong (Hong Kong, China). Female C57BL/6 mice (age, 10 weeks, 22 ± 2 g) were used in the experiments. The mice were housed in a pathogen-free environment at 23 ± 2 °C under a 12-h/12-h light/dark cycle. All experiments and animal care procedures in this study were performed according to the Guide to Animal Use and Care of the University of Macau and were approved by the Animal Ethics Committee of Institute of Chinese Medical Science, University of Macau. B16-F10 cells (5 × 10^4^ cells in 0.1 ml phosphate-buffered saline, PBS) were transplanted subcutaneously (s. c.) into mice on day 0. Tumor-bearing mice in treatment group received FAE treatment by oral gavage on day 0 and every two days after that till the endpoint. The tumor model group and control group (without tumor) only received normal drinking water. The length and width of the tumors were measured with a sliding caliper. The tumor size (S) was estimated according to the formula S = L × W^2^/2, where L is length, W is width. Tumor size was monitored twice a week. Body weight and mortality of the mice were monitored every two days. Mice were sacrificed when the tumor size reaches 10% of the body weight. Blood was collected from the heart of each mouse on the sacrificed day. After centrifugation at 1300 g for 10 min, the serum was isolated and stored at −80 °C for detection.

### Sample preparation for metabolic profiling analysis

The processing steps of the serum samples were modified according to the protocol reported by Dunn *et al*.^45^. For the pretreatment of the serum samples, serum sample was thawed on ice at 4 °C. Then, 300 μl cold methanol was added to 100 μl serum. The mixture was vortexed for 15 s and centrifuged at 13000 g for 15 min. Next, the supernatant was transferred to another centrifuge tube and then freeze-dried on a Nitrogen evaporator N-EVAP 112 (Organomation Associates, Inc., Berlin, MA, USA) with no heating. 50 μl of water was added to dried samples, vortex for 15 s and centrifuge at 13000 g for 15 min. In order to monitor the repeatability of sample analysis, quality control (QC) samples were added into the analysis sequence. The QC sample was prepared by equally mixing the tested serum samples. Transfer 40 μl of supernatant to the sample vials, and stored at 4 °C pending UPLC/Q-TOF MS analysis.

### Metabolic profiling

Liquid chromatography was performed using a Waters ACQUITYTM ultra performance liquid chromatography (UPLC, Waters Corp., Milford, MA, USA). Five μL aliquot of each sample was injected into an ACQUITY UPLC HSS T3 C18 column (100 mm × 2.1 mm I.D., 1.8 μm) maintained at 45 °C. The mobile phase consisted of a linear gradient system of 0.1% formic acid in water (solution A) and 0.1% formic acid in acetonitrile (solution B), 0–1 min, 1% B; 1–7 min, 1–72% B; 7–10 min, 72% B; 10–17 min, 72–100% B; 17–19 min, 100% B; 19–21 min, 100%-1% B; 21–24 min, 1% B. The flow-rate was 0.45 mL/min.

Mass spectrometry was performed using a SYNAPT G2-Si high-definition mass spectrometer (Waters Corp., Milford, MA, USA) operated using both the positive (ESI+) and negative (ESI−) ion modes. Source temperature was set at 120 °C with a cone gas flow of 10 L/hr. Meanwhile, the desolvation gas temperature was 450 °C with gas flow of 900 L/hr. The capillary voltage was set to 3.0 kV (ESI+) or 2.5 kV (ESI−), sampling cone voltage was set to 40 V. The extraction cone voltage was 4.0 V, the TOF acquisition rate was 0.1 s/scan. MS/MS data were collected for all the ions observed in the preceding MS scan. In order to ensure the accuracy and reproducibility of Q-TOF MS, the leucine enkephalin calibrant solution at the concentration of 200 ng/mL was used as the lock mass in positive ion mode (m/z 556.2771) and negative ion mode (m/z 554.2615). A full scan mass range from m/z 50 to m/z 1200 was scanned.

### Data Processing and Analysis

The raw data were imported to Markerlynx software (Waters Corporation, MA, USA) for peak detection and alignment to obtain a peak list containing the retention time, m/z, and peak area of each sample. The peak area was normalized to an internal standard for further statistical analysis. Then, the resultant data matrices were introduced into the SIMCA-P software (Umetrics AB, Umea, Sweden) for multivariate pattern recognition analysis, including PCA, PLS-DA and OPLS analysis. From the S-plot of OPLS, the clustering information and potential markers of control group, tumor model group and treatment group were acquired. Significantly changed potential markers among all the groups were chosen according to the VIP values (>3) based on their contribution to the variation and correlation within the data set. To support the potential biomarkers identification and further understanding of this study, PubChem (http://ncbi.nim.nih.gov), MassBank (http://www.massbank.jp), Human Metabolome Database (HMDB, http://www.hmdb.ca), METLIN (http://metlin.scripps.edu), KEGG (http://www.genome.jp/kegg) and MetaboAnalyst (http://www.metaboanalyst.ca) were queried.

### Cell culture

Murine melanoma B16-F10 cell line and NIH-3T3 cell line were purchased from the Cell Bank of Type Culture Collection of Chinese Academy of Sciences (Shanghai, China). Cells were cultured in basic medium supplemented with 10% heat-inactivated fetal bovine serum, 100 U/ml penicillin, and 100 μg/ml streptomycin at 37 °C in a humidified atmosphere of 5% CO_2_. The medium was changed every other day. Cells were plated at a density of 5 × 10^5^ cells in 100 mm dish. After 16~20 h incubation, the cells were treated with a series of concentrations of FAE for 24 h at 37 °C.

### Western blotting

The method of western blotting was same with the previous paper^44^. Protein was extracted from tumor tissues and cells using RIPA buffer. NIH-3T3 cells were used as normal group in the *in vitro* experiment. β-actin was used as the internal reference.

### Statistical analysis

One-way ANOVA analysis with Tukey post hoc was performed to compare differences in variables of control, tumor model and FAE treatment groups using GraphPad Prism software (La Jolla, CA, USA). Statistical significance was accepted at the level of *p* < 0.05.

## Additional Information

**How to cite this article**: Bao, J. *et al*. Anti-melanoma activity of Forsythiae Fructus aqueous extract in mice involves regulation of glycerophospholipid metabolisms by UPLC/Q-TOF MS-based metabolomics study. *Sci. Rep.* **6**, 39415; doi: 10.1038/srep39415 (2016).

**Publisher's note:** Springer Nature remains neutral with regard to jurisdictional claims in published maps and institutional affiliations.

## Figures and Tables

**Table 1:** The identified serum metabolites with significant changes in content.

Var ID	Ret. Time	m/z Determined	C/M VIP	C vs M	M/F VIP	M vs F	MS/MS	Ion Form	Molecular Formula	Metabolite identification
P1624	7.72	496.3366	4.40227	↓	6.02723	↑	184.0408	[M+H]^+^	C_24_H_50_NO_7_P	LysoPC (16:0)
P627	7.62	1063.7493	3.39352	↓	3.19738	↑	520.3394, 184.0415	[2M+Na]^+^	C_26_H_50_NO_7_P	LysoPC (18:2)
P1696	8.17	522.3577	5.28938	↓	9.1499	↑	184.0391	[M+H]^+^	C_26_H_52_NO_7_P	LysoPC (18:1)
P1703	8.85	524.3699	4.37317	↓	6.16404	↑	184.039	[M+H]^+^	C_26_H_54_NO_7_P	LysoPC (18:0)
P1757	7.60	544.3415	7.8621	↓	8.50015	↑	520.3417, 184.0421	[M+H]^+^	C_28_H_50_NO_7_P	LysoPC (20:4)
P1820	7.59	568.3453	6.88901	↓	6.8384	↑	520.3417, 184.0419	[M+H]^+^	C_30_H_50_NO_7_P	LysoPC (22:6)
P2154	15.97	782.6029	3.38677	↑	3.14485	↓	184.0386	[M+H]^+^	C_44_H_80_NO_8_P	PC (36:4)
P1390	8.50	786.6342	3.15525	↑	3.54181	↓	184.0389	[M+H]^+^	C_44_H_84_NO_8_P	PC (36:2)
P2063	12.44	758.5992	3.65152	↑	3.85306	↓	184.0392	[M+H]^+^	C_43_H_84_NO_7_P	PE (38:1)
P2253	15.23	806.6061	6.07016	↑	6.21881	↓	184.0388	[M+Na]^+^	C_45_H_86_NO_7_P	PE (40:2)
P135	2.62	120.0454	3.64872	↓	3.11635	↑	103.019	[M+H]^+^	C_4_H_9_NO_3_	L-Threonine
P524	2.98	188.0377	7.86141	↓	5.53546	↑	143.0372, 115.0186	[M+Na]^+^	C_8_H_7_NO_3_	Formylanthranilic acid
N138	3.39	336.0458	3.91943	↑	3.7903	↓	271.9555	[M−H]^−^	C_11_H_16_NO_9_P	Nicotinic acid mononucleotide
P720	5.13	1089.6114	4.03286	↓	4.25722	↑	567.2073, 258.1142	—	—	unidentified
P843	6.13	250.1466	4.82278	↑	5.36538	↓	184.0384	—	—	unidentified
P1094	5.98	307.0693	3.40107	↑	3.78951	↓	184.0385, 159.0811	—	—	unidentified
P2307	3.48	820.9112	6.88683	↓	5.43863	↑	505.2203, 129.0666	—	—	unidentified

Notes: LysoPC, lysophosphatidylcholine; PC, phosphatidylcholine; PE, phosphatidylethanolamine; VIP, variable importance in projection; C, control group; M, tumor model group; F, FAE treatment group.

**Table 2:** Summary of pathway analysis.

	Pathway Name	Total	Hits	p	−log (p)	Holm p	FDR	Impact
a	Glycerophospholipid metabolism	30	3	1.64E-04	8.7136	0.013476	0.013476	0.275
b	Linoleic acid metabolism	6	1	0.025182	3.6816	1	0.95013	0
c	alpha-Linolenic acid metabolism	9	1	0.037574	3.2814	1	0.95013	0
d	Nicotinate and nicotinamide metabolism	13	1	0.053892	2.9208	1	0.95013	0.17262
e	Glycosylphosphatidylinositol (GPI)-anchor biosynthesis	14	1	0.057935	2.8484	1	0.95013	0.0439
f	Glycine, serine and threonine metabolism	31	1	0.1245	2.0835	1	1	0
g	Arachidonic acid metabolism	36	1	0.14331	1.9427	1	1	0
h	Tryptophan metabolism	40	1	0.15812	1.8444	1	1	0.00642
i	Aminoacyl-tRNA biosynthesis	69	1	0.25923	1.35	1	1	0

**Table 3:** Pathway analysis of the identified metabolites.

Metabolite identification	HMDB ID	KEGG ID	Relative pathway	Main pathway
LysoPC (16:0)	HMDB10382	C04230	a	Glycerophospholipid metabolism
LysoPC (18:2)	HMDB10386	C04230	a	Glycerophospholipid metabolism
LysoPC (18:1)	HMDB02815	C04230	a	Glycerophospholipid metabolism
LysoPC (18:0)	HMDB10384	C04230	a	Glycerophospholipid metabolism
LysoPC (20:4)	HMDB10395	C04230	a	Glycerophospholipid metabolism
LysoPC (22:6)	HMDB10404	C04230	a	Glycerophospholipid metabolism
PC (36:4)	HMDB08014	C00157	a, b, c, g	Glycerophospholipid metabolism
PC (36:2)	HMDB08071	C00157	a, b, c, g	Glycerophospholipid metabolism
PE (38:1)	HMDB09249	C00350	a, e	Glycerophospholipid metabolism
PE (40:2)	HMDB09577	C00350	a, e	Glycerophospholipid metabolism
L-Threonine	HMDB00167	C00188	i	Glycine, serine and threonine metabolism
Formylanthranilic acid	HMDB04089	C05653	h	Tryptophan metabolism
Nicotinic acid mononucleotide	HMDB01132	C01185	d	Nicotinate and nicotinamide metabolism

Notes: (a – i), metabolic pathways listed in Table 2.
